# GC/MS and LC/MS serum metabolomic analysis of Chinese LN patients

**DOI:** 10.1038/s41598-024-52137-w

**Published:** 2024-01-17

**Authors:** Wei Wang, Jun Kou, Jie Long, Tao Wang, Mingmei Zhang, Meng Wei, Qingyun Xie

**Affiliations:** 1 Department of Orthopedics, General Hospital of Western Theater Command, Rongdu Avenue No. 270, Chengdu, 610000 People’s Republic of China; 2 Department of Ultrasound Children’s Hospital of Chongqing Medical University, National Clinical Research Center for Child Health and Disorders, Ministry of Education Key Laboratory of Child Development and Disorders (Chongqing Key Laboratory of Pediatrics), Chongqing, 400010 China; 3 Department of Nephrology, Honghui Hospital, Xi’an Jiaotong University College of Medicine, No.555 Youyi East Road, Beilin District, Xi’an, 710054 Shaanxi People’s Republic of China; 4 Department of Rheumatism and Immunology, The General Hospital of Western Theater Command, Tianhui Road 270, Chengdu, 610000 People’s Republic of China

**Keywords:** Immunological disorders, Kidney diseases, Rheumatic diseases

## Abstract

China, being a densely populated nation, faces a substantial economic burden due to a high incidence of lupus nephritis (LN) cases. The concealed onset of LN has resulted in many individuals have missed the optimal timing for treatment. The aim of the research is to study the serum metabolomics of Chinese LN patients using gas chromatography (GC)/mass spectrometry (MS) and liquid chromatography (LC)/MS to identify potential diagnostic markers. Fifty LN patients and fifty normal controls, matched for Body Mass Index (BMI) and age, were selected. Serum analysis was conducted using GC/MS and LC/MS, followed by multivariate statistical analysis. Various multidimensional analyses, including principal component analysis, partial least squares discrimination analysis, and orthogonal partial least squares discrimination analysis, along with one-dimensional analyses such as t-tests, were performed. Metabolites with variable importance in projection value > 1 and a p-value < 0.05 were considered critical biomarkers for LN. Furthermore, identified biomarkers delineated relevant metabolic pathways, and a metabolic pathway map was obtained from the database. Forty-one metabolites were identified as potential LN biomarkers, primarily associated with immune regulation, energy metabolism, intestinal microbial metabolism, renal damage, and oxidative stress. The potential for diagnosing LN and other diseases through metabolomics is demonstrated. Future research should explore larger sample sizes, metabolomic comparisons across different diseases and health states, and integration of metabolomics with clinical diagnostics. Such studies will enhance the understanding of metabolomics in medical diagnosis and provide robust support for its practical application.

## Introduction

Systemic lupus erythematosus (SLE) is a condition where the immune system targets healthy cells and tissues throughout the body. Key clinical signs range from mild fatigue and joint pain to severe, life-threatening organ damage^1^. Numerous studies indicate that immune complexes activating the classical complement pathway are the primary cause of organ and tissue injuries in SLE. Aspects such as antibody production and elimination, circulation and tissue deposition of immune complexes, and the activation of complement and cytokines are widespread in SLE^2^. Lupus nephritis (LN) is a crucial clinical aspect of SLE, with renal involvement exhibiting manifestations like proteinuria, erythrocyte urine, leukocyturia, tubular urine, reduced glomerular filtration function, and diminished renal tubular function. The severity of renal damage significantly influences the prognosis of SLE, and renal involvement is a leading cause of death in SLE^3^. Evidence suggests that non-European populations experience more severe symptoms than their European counterparts, with higher rates of cardiovascular disease, renal involvement, and tissue injury^4^. In East Asian patients, the onset age of LN is earlier compared to other ethnic groups^5^. China, being heavily populated, harbors a substantial number of LN patients facing disease-related challenges and economic burdens. Early diagnosis and tailored intervention strategies for clinical remission or low disease activity present opportunities to minimize damage and enhance long-term outcomes^6^. Currently, renal biopsy serves as the gold standard for diagnosing LN^7^; however, many patients are diagnosed post-onset, posing challenges for early diagnosis and intervention.

Metabonomics, a widely used experimental system biology tool, holds significant potential in the study of metabolites across plant, microorganism, and mammalian research^8^. Metabolism is intricately linked to nearly all cellular processes, and disturbances in cell physiology often result in altered metabolic footprints^9^. Technological advancements, particularly in mass spectrometry (MS) and data analysis tools, have greatly facilitated the progress of metabolomics. MS-based metabonomics enables the quantification of numerous small molecules in body fluids or tissues in a single step, offering substantial promise in comprehending the pathogenesis of various diseases and developing novel methods for early diagnosis and treatment monitoring^10^.

Gas chromatography (GC) and liquid chromatography (LC) are widely employed platforms for metabolomics analysis. GC/MS is commonly utilized for analyzing volatile organic compounds, lipids, and derivable molecules due to its high repeatability and robustness. However, it has limitations in observing non-volatile compounds that cannot be derivatized and large or thermally unstable compounds^11^. In contrast, LC/MS offers more advantages for nonvolatile compounds and large or thermally unstable compounds, making it the most comprehensive method in metabolomics research. Simultaneous application of GC/MS and LC/MS methods in metabolomics research enhances our understanding of metabolic spectrum changes^12^. Untargeted metabonomics, which analyzes all detectable metabolites without relying on pre-specified categories, can identify potential biomarkers for diseases^13^.

Existing literature reveals that metabolomics research on lupus nephritis (LN) patients typically employs a single method, resulting in a relatively narrow identification area of metabolites and small participant numbers in most studies. Currently, there is no metabolomic study on LN patients in the Chinese population using both GC/MS and LC/MS. This study focuses on determining serum metabolites in LN patients and normal subjects through untargeted GC/MS and LC/MS. The objective is to conduct a metabolomic analysis, employing multidimensional and one-dimensional approaches to identify metabolite differences between the two groups and screen potential biomarkers for early LN detection and treatment monitoring.

## Material and methods

### Participants

A total of 50 patients diagnosed with LN in our hospital from August 2017 to May 2018 were included in this study. Inclusion criteria were based on the ACR1997 standard^14^ for LN diagnosis. Exclusion criteria included: (1) Evident damage to the liver, brain, lungs, blood, or other systems; (2) Participants with health complications potentially impacting metabolism; (3) Participants who underwent treatments or took medications affecting metabolism; (4) Individuals with substance addiction. Additionally, 50 healthy individuals, matched in terms of age, gender, and body mass index (BMI), were recruited as the control group. The same exclusion criteria applied to the control group, ensuring they had no specific diseases or abnormal physical conditions. All volunteers were required to carefully review and sign informed consent forms. The clinical study received approval from the hospital ethics committee (Western Theater General Hospital).

### Sample collection and processing

Whole blood from each patient was collected in an untreated sterile non-anticoagulant tube on an empty stomach in the morning. After a 30-min standing period, the blood was centrifuged at 1600*g* at room temperature (22 °C) for 15 min. The upper serum was collected, and 300 μL of each serum was frozen in liquid nitrogen for 30 s and stored at − 80 °C for subsequent analysis.

For sample preparation, the stored samples at − 80 °C were thawed at room temperature, and 80 μL samples were taken to a 1.5 ml Eppendorf tube. Internal standard (L-2-chlorophenylalanine, 0.3 mg/ml, methanol configuration) at 10 μL was added, followed by vortex oscillation for 10 s. Subsequently, 240 μL of methanol–acetonitrile (V:V = 2:1) mixed solution was added, and vortex shaking was performed for 1 min. Ultrasonic extraction in an ice water bath for 5 min was carried out, followed by standing at − 19 °C for 10 min. Centrifugation for 10 min (12,000 rpm, 4 °C) was done, and 150 μL of the supernatant was transferred into a glass derivatization bottle. The sample was then evaporated using a freeze concentration centrifugal dryer. For the next step, 80 μL of methoxyamine hydrochloride pyridine solution (15 mg/ml) was added to the glass derivatization vial. After vortex shaking for 2 min, an oximation reaction was conducted in a shaking incubator at 37 °C for 90 min. The sample was taken out, and 80 μL of BSTFA (containing 1% TMCs) derivatization reagent and 20 μL of n-Hexane were added. After vortex shaking for 2 min, the reaction was carried out at 70 °C for 60 min. Following this, the sample was left at room temperature for 30 min before undergoing GC–MS metabolomics analysis.

Moreover, 100 μL of serum thawed at room temperature was used for LC–MS analysis. Internal standards (L-2-chlorophenylalanine, 0.3 mg/ml; C-17, 0.01 mg/ml, both in methanol configuration) at 10 μL each were added, followed by vortex oscillation for 10 s. Subsequently, 300 μL of protein precipitant methanol–acetonitrile (V:V = 2:1) was added, and vortex oscillation was performed for 1 min. Ultrasonic extraction for 10 min in an ice water bath and standing at − 20 °C for 30 min were conducted. After centrifugation for 10 min (13,000 rpm, 4 °C), 200 μL of the supernatant was extracted with a syringe. Using a 0.22 μm organic phase pinhole filter, the supernatant was transferred to an LC injection vial and stored at − 80 °C until LC–MS analysis.

### Metabolite measurement and multivariate data analysis

The detailed procedures for metabolite measurement and multivariate data analysis have been previously outlined in our publications^15^.

### Find key biomarkers and analysis metabolic pathway

A combination of multi-dimensional and one-dimensional analyses was employed to identify differential metabolites between groups. The screening criteria included a Variable Importance for Projection (VIP) value exceeding 1 for the first principal component of the Orthogonal Partial Least Squares Discrimination Analysis (OPLS-DA) model and a p-value below 0.05. The fold change (FC) for the differential metabolites in the two groups was calculated as the ratio of the average content of the metabolite in the two groups.

Additionally, volcanic maps and hierarchical clustering of related metabolites were generated. The volcanic map visualized p-values and fold change values, aiding in the identification of differential metabolites. Hierarchical clustering provided an intuitive representation of the relationship between samples and the expression differences of metabolites. Subsequently, utilizing the Kyoto Encyclopedia of Genes and Genomes (KEGG), we identified metabolic pathways associated with these key metabolites. Verification of their pathological relationship with lupus nephritis (LN) was performed through relevant literature, ultimately yielding a metabolic pathway map. This map facilitates understanding the mechanism of metabolic pathway changes in different samples.

### Statistical methods

Results are presented as means ± standard deviations. The normality and homogeneity of variance for all data were assessed using the Kolmogorov–Smirnov test. A two-sided Student t-test was employed for comparative analysis between two groups, while a one-way analysis of variance explained differences among more than two groups. The significance threshold was set at P < 0.05. Statistical analyses were performed using the Statistical Package for the Social Sciences, version 24.0 (SPSS, Chicago, IL).

### Ethics approval and consent to participate

All procedures involving human participants in this study were carried out in accordance with the 1964 Helsinki Declaration and its subsequent amendments or similar ethical standards. This clinical study was approved by the ethics committee of our hospital (General Hospital of Western Theater Command, ID number: 2021-XZYG-B05). Informed consent was obtained from all subjects and their legal guardians.

## Results

### Participants

A total of 50 LN patients and 50 normal subjects were included in the study. Table 1 presents the matched BMI, age, and sex ratio between the healthy control group and the case group. Statistical data analysis confirmed conformity to the normal distribution.

**Table 1:** The characteristics of LN group and healthy control group.

	LN (number or mean ± SD)	HCG (number or mean ± SD)	P*
Total number	50	50	–
Sex (female/male)	48\2	44\6	–
Age (year)			
Range	21–69	18–63	
Average	49.92 ± 11.59	43.72 ± 11.39	0.72
BMI (kg/m^2^)	25.24 ± 6.25	24.86 ± 4.65	0.73

*BMI* body mass index, *LN* lupus nephritis, *HCG* healthy control group, *SD* standard deviation.

*Calculated by Student’s t-tests for continuous variables and Chi2 tests for categorical variables between LN patients and healthy controls.

### Untargeted GC/MS and LC/MS analysis of samples

Comprehensive metabolomic analysis of serum samples from both groups was conducted. Following multivariate analysis and considering metabolite VIP, FC, and P values, 41 metabolites were identified as potential LN biomarkers (Table 2). Specific metabolites detected by GC/MS and LC/MS are detailed in Table 2.

**Table 2:** Summary of potential biomarkers of LN by serum GC/MS AND LC/MS analysis.

Metabolite	Status^a^	VIP value^b^	FC^c^	P^※^	Data origin
3-Hydroxybutyric acid	↓	1.004	0.674	0.035	GC–MS
Asparagine	↑	1.066	1.272	< 0.001	GC–MS
Beta-alanine	↑	1.187	2.194	0.005	GC–MS
Citric acid	↓	1.218	0.807	0.002	GC–MS
Creatinine	↑	1.635	3.853	0.014	GC–MS
Cystathionine	↓	2.011	0.738	< 0.001	GC–MS
d-Erythro-sphingosine	↑	1.568	1.993	0.006	GC–MS
Glutamate	↑	1.934	3.132	< 0.001	GC–MS
Glutamine	↑	1.909	2.023	< 0.001	GC–MS
Glycine	↓	1.075	0.806	0.008	GC–MS
Guanosine	↓	1.032	0.434	< 0.001	GC–MS
Histidine	↓	1.243	0.692	< 0.001	GC–MS
Hydroquinone	↓	1.171	0.489	0.001	GC–MS
Hydroxoproline	↑	1.047	1.665	< 0.001	GC–MS
Linoleic acid	↑	2.11	1.996	< 0.001	GC–MS
Lysine	↓	1.421	0.764	< 0.001	GC–MS
Ornithine	↓	1.516	0.577	< 0.001	GC–MS
Palmitic acid	↑	2.149	1.797	< 0.001	GC–MS
Phenylalanine	↓	2.154	0.706	< 0.001	GC–MS
Piceatannol	↓	1.705	0.765	< 0.001	GC–MS
Pseudo uridine	↑	1.231	4.635	0.002	GC–MS
Serine	↓	2.111	0.659	< 0.001	GC–MS
Succinic acid	↓	1.436	0.778	< 0.001	GC–MS
Threonine	↓	1.27	0.77	< 0.001	GC–MS
Tryptophan	↓	1.093	0.81	0.003	GC–MS
Tyrosine	↓	1.005	0.868	0.013	GC–MS
Udp-glucuronic acid	↑	1.845	8.431	0.001	GC–MS
Valine	↓	2.064	0.708	< 0.001	GC–MS
Xylitol	↑	1.539	1.531	0.006	GC–MS
l-Carnitine	↑	2.237	2.322	< 0.001	LC–MS
d-Glucose	↑	2.649	1.481	< 0.001	LC–MS
TG	↑	1.191	2.02	< 0.001	LC–MS
Ceramide	↑	2.431	99,860,110.64	0.001	LC–MS
PS	↓	3.91	0.331	< 0.001	LC–MS
PI	↓	1.351	0.128	< 0.001	LC–MS
PE	↓	1.096	0.135	< 0.001	LC–MS
PG	↓	2.471	0.0004	< 0.001	LC–MS
PA	↓	3.321	0.106	< 0.001	LC–MS
SM	↓	1.026	0.368	< 0.001	LC–MS
PC	↓	3.272	0.88	0.035	LC–MS
Cholesterol	↓	1.157	3.83	< 0.001	LC–MS

*TG* triacylglycerol, *PS* phosphatidylserine, *PI* phosphatidylinositol, *PE* phosphatidylethanolamine, *PG* phosphatidylglycerol, *PA* phosphatidic acid, *SM* sphingomyelin, *PC* phosphatidylcholine, *FC* fold change, *VIP* variable importance for projection,

^a^Relative concentrations compared to healthy controls: ↑ = up-regulated, ↓ = down-regulated, ^b^VIP value were obtained from OPLS-DA analysis, ^c^Fold change between LN patients and healthy controls group, ^※^P value determined from Student t test.

### Multivariate data analysis base on MS data

**Figure 1:**
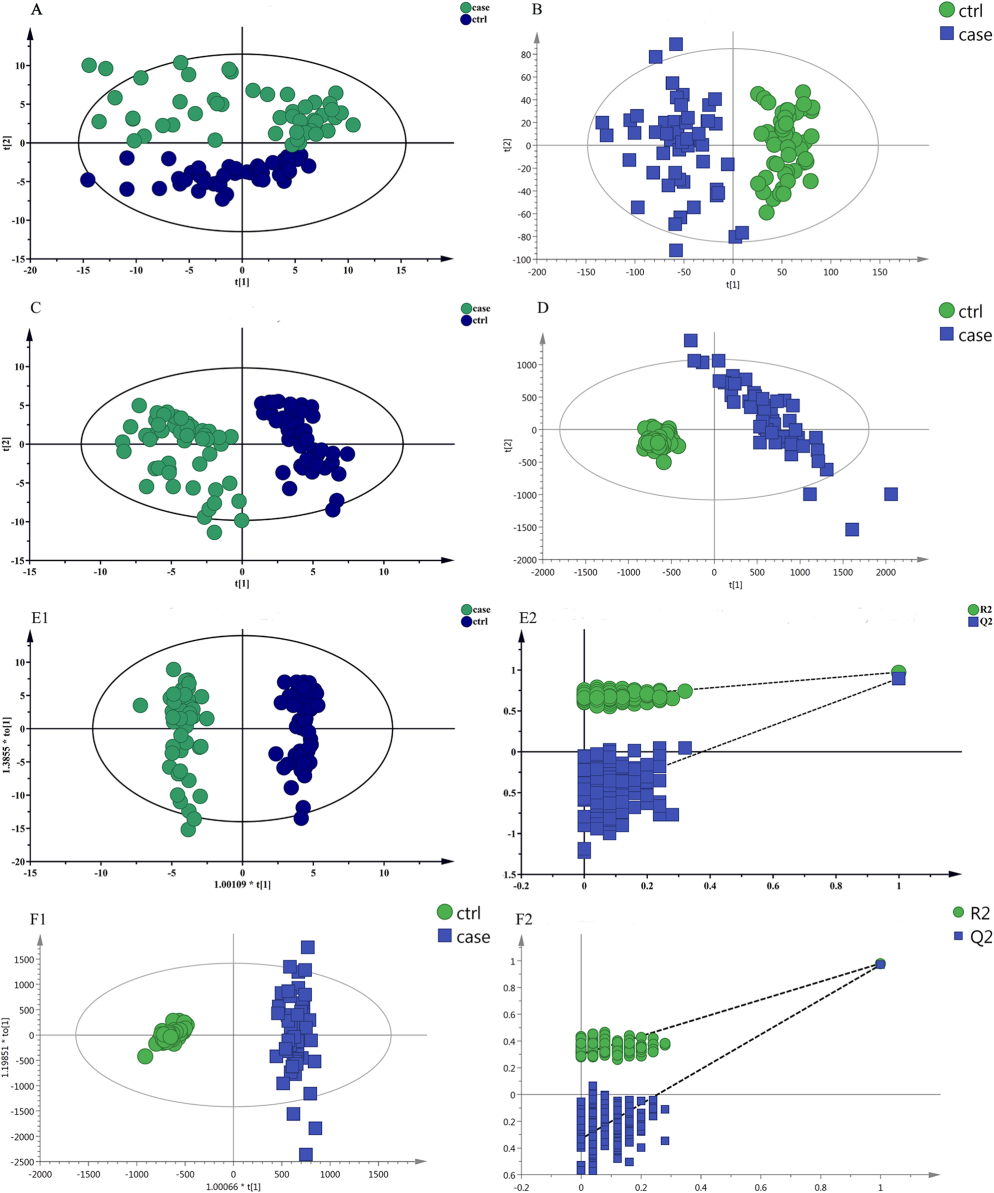
Multivariate date analysis of date from serum between the case group (Green) and healthy control group (Blue) base on GC/MS AND LC/MS. (**A**) PCA score plots based on the GC–MS. (**B**) PCA score plots based on the LC–MS. (**C**) PLS-DA score plots based on the GC–MS. (**D**) PLS-DA score plots based on the LC–MS. (**E1**,**E2**) OPLS-DA score plots (left panel) and Statistical validation of the corresponding OPLS-DA model by permutation analysis (right panel) based on the GC–MS. (**F1**,**F2**) Statistical validation of the corresponding OPLS-DA model by permutation analysis (right panel) based on the LC–MS. The two coordinate points are relatively far away on the score map, indicating that there is a significant difference between the two samples, and vice versa. The elliptical region represents a 95% confidence interval.

Principal component analysis (PCA) demonstrated evident separation between the two sample groups, indicating substantial metabolic differences (Fig. 1A,B). Significant differences were also observed in partial least squares discrimination analysis (PLS-DA) (Fig. 1C,D). The high values of model interpretation rate (R2Y) and prediction rate (Q2), with R2Y and Q2 at 0.974 and 0.905 (GC–MS), 0.98 and 0.969 (LC–MS) respectively, indicated the effective explanatory and predictive capabilities of the PLS-DA model. In orthogonal partial least squares discrimination analysis (OPLS-DA), R2Y and Q2 were 0.974 and 0.9 (GC–MS), 0.98 and 0.97 (LC–MS), respectively, confirming significant differences between the two groups (Fig. 1E1,F1). Response permutation testing in OPLS-DA further verified the absence of overfitting (Fig. 1E2,F2).

### Potential biomarkers and pathway analysis

**Figure 2:**
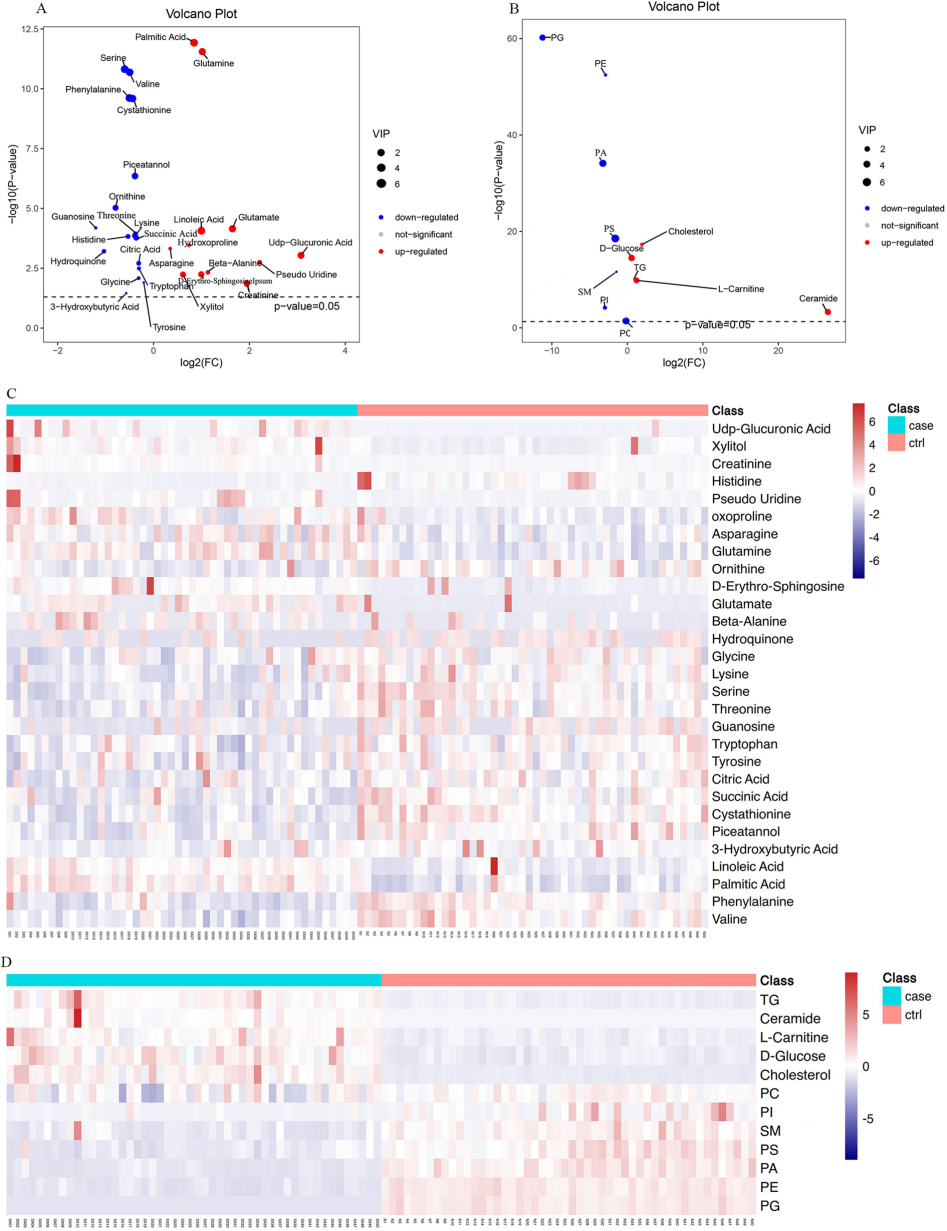
Volcano plot and hierarchical clustering based on the GC/MS AND LC/MS of serum metabolites obtained from the case group (blue) and healthty control group (red). (A) Volcano plot based on the GC–MS. (**B**) Volcano plot based on the LC–MS. (**C**) Hierarchical clustering based on the GC–MS. (**D**) Hierarchical clustering based on the LC–MS. In (**A**) and (**B**), the blue dot represent metabolite with an downward trend, red represents metabolites with an upward trend, and the gray origin represents that the change of metabolites is not obvious. The area size of the point is related to the VIP value. In the (**C**) and (**D**), the color from blue to red illustrate that metabolites expression abundance is low to high in hierarchical clustering. *TG* triacylglycerol, *PS* phosphatidyl-serine, *PI* phosphatidylinositol, *PE* phosphatidylethanolamine, *PG* phosphatidyl-glycerol, *PA* phosphatidic acid, *SM* sphingomyelin, *PC* phosphatidylcholine.

**Figure 3:**
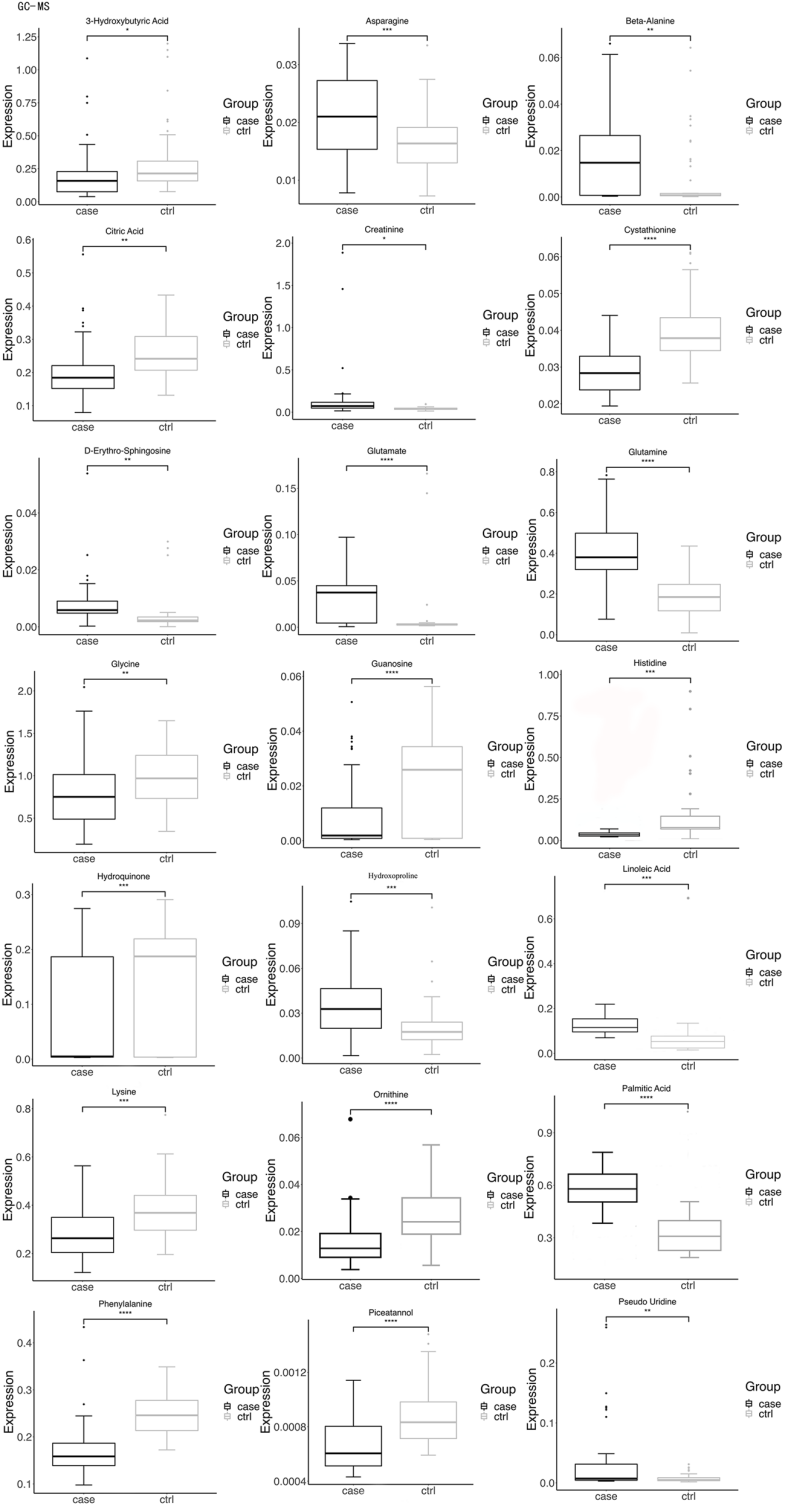

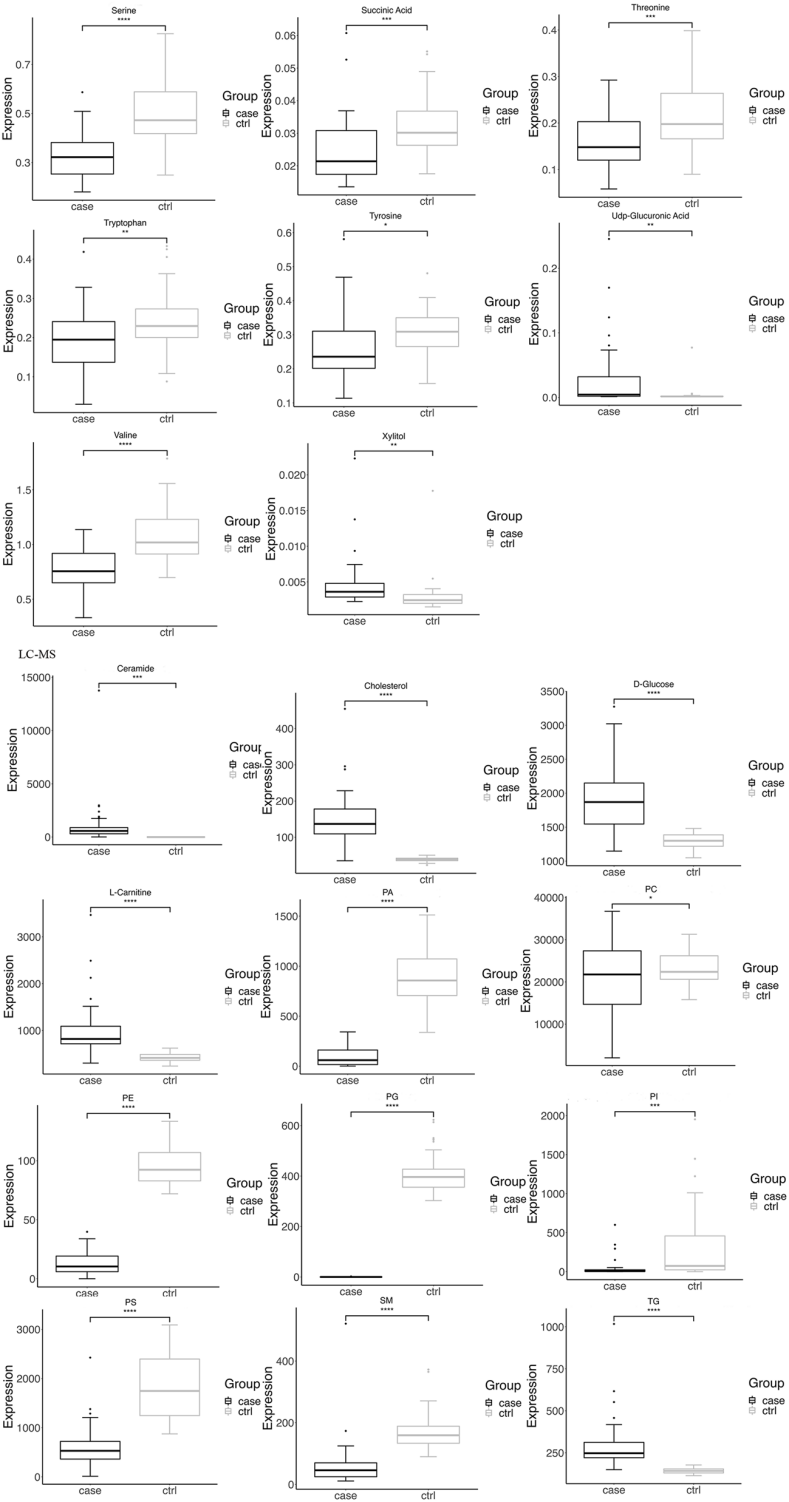
Box-and-whisker plots showing the relative levels of selected potential biomarkers for LN. The black box on the left represents the case group and the grey box on the right represents the healthy control group. Horizontal line in the middle portion of the box, median; bottom and top boundaries of boxes, lower and upper quartile; whiskers, 5th and 95th percentiles. *p < 0.05, **p < 0.01, ***p < 0.001, ****p < 0.0001. *TG* triacylglycerol, *PS* phosphatidylserine, *PI* phosphatidylinositol, *PE* phosphatidylethanolamine, *PG* phosphatidylglycerol, *PA* phosphatidic acid, *SM* sphingomyelin, *PC* phosphatidylcholine.

**Figure 4:**
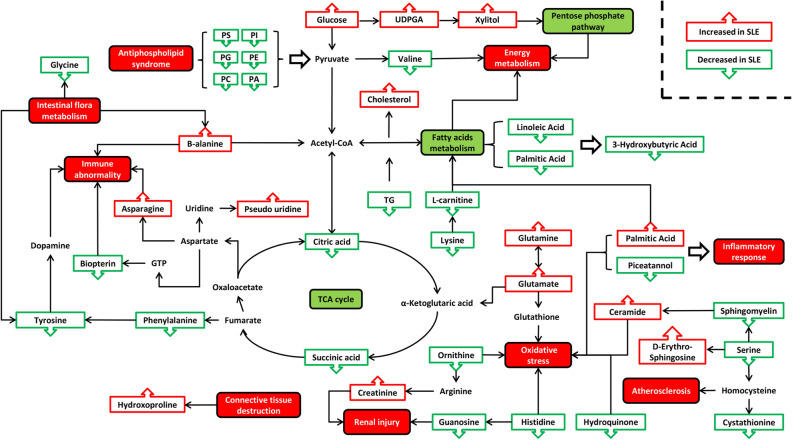
Altered metabolic pathways for the most relevant distinguishing metabolites (potential biomarkers) between the LN group and healthy control group. The metabolites with red border were up-regulated in case group, whereas with green border indicated metabolites that were down-regulated. *TG* triacylglycerol, *PS* phosphatidylserine, *PI* phosphatidylinositol, *PE* phosphatidylethanolamine, *PG* phosphatidylglycerol, *PA* phosphatidic acid, *SM* sphingomyelin, *PC* phosphatidylcholine, *TCA* the tricarboxylic acid cycle.

Box plots (Fig. 2), volcano plots, and hierarchical clustering (Fig. 3) of selected metabolites revealed significant differences between groups. Database searches and literature consultations indicated that these metabolites were primarily associated with immune regulation, energy metabolism, intestinal microbial metabolism, renal damage, and oxidative stress. The relationship between these metabolites was visually represented through a metabolic pathway map of markers with significant differences (Fig. 4).

## Discussion

Metabonomics assesses various compounds within living systems, showing promise for diagnosis, early disease detection, treatment selection, and treatment monitoring^10^. In our study, we demonstrated significant metabolic differences in the serum of LN patients compared to that of normal individuals. These differences primarily involve immune regulation, energy metabolism, intestinal microbial metabolism, renal damage, and oxidative stress.

Our investigation revealed an elevated concentration of asparagine in LN serum. Some reports propose that asparaginase can regulate the B-lymphocyte response mediated by T-lymphocytes through asparagine hydrolysis, thereby inhibiting the immune response and exerting an anti-inflammatory effect^16^. Consequently, asparaginase is considered to hold therapeutic potential for autoimmune diseases. In LN patients' serum, we observed a substantial decrease in the concentrations of phenylalanine and tyrosine. Phenylalanine, an essential amino acid, generates tyrosine via phenylalanine hydroxylase, and dopamine is a crucial metabolite of tyrosine. Studies indicate a significant increase in dopamine content in autoimmune disease patients, including those with SLE, suggesting the involvement of the dopamine system in autoimmune inflammation and immune regulation^17^. Thus, the reduced levels of phenylalanine and tyrosine may be linked to the heightened downstream content of dopamine. Pseudouridine, a component of tRNA synthesized by the human body, can disrupt RNA's ability to stimulate the immune system^18^. We observed a significant increase in pseudouridine levels in LN patients' serum, potentially contributing to the immune system disorder in LN. Additionally, the content of biopterin in LN patients' serum exhibited a marked decrease. Other studies reported similar findings in patients with SLE, rheumatoid arthritis (RA), and multiple sclerosis (MS), associating this abnormal change with excessive autoimmune responses^19,20^. However, the specific mechanism remains unclear.

In this study, we observed a significant increase in triglycerides, cholesterol, and certain long-chain fatty acids (linoleic acid and palmitic acid) in LN serum. Simultaneously, the concentration of L-Carnitine, responsible for transporting fatty acids into mitochondria for β-Oxidation, was notably reduced in LN serum. Furthermore, the downstream product of β-Oxidation, 3-hydroxybutyric acid, also exhibited a significant decrease. Numerous prior studies have also highlighted abnormal lipid metabolism in LN patients, with a notable rise in triglyceride and cholesterol levels. This phenomenon contributes to an increased prevalence of atherosclerosis in LN patients, reaffirmed by our study^21,22^. Alanine, an intermediate metabolite, directly facilitates coenzyme A (CO-A) biosynthesis and plays a crucial role in energy metabolism. The substantial increase in its concentration in LN serum may result from CO-A synthesis pathway obstruction, correlating with diminished energy production in LN. Additionally, LN patients displayed a significant increase in serum glucose levels. Concurrently, citric acid, a downstream product of glucose energy metabolism and a key metabolite in the tricarboxylic acid (TCA) cycle, exhibited a marked decrease. Similarly, the concentration of succinic acid, a key intermediate in the TCA cycle, also decreased significantly in LN serum. These abnormal changes in three metabolites may indicate disrupted energy metabolism in LN patients, signifying a considerable weakening of energy derived from glucose. These results align with findings from two previous related studies^23,24^. As the aerobic metabolic pathway of glucose weakens, the glucuronic acid pathway, initially a minor component of glucose metabolism, may become overactivated. In the glucuronic acid pathway, glucose transforms into UDPGA, and subsequent reactions yield xylitol, which then enters the pentose phosphate pathway for metabolism^25^. In our study, we observed a significant increase in the contents of UDPGA and xylitol in LN patients' serum. This finding not only affirms the disruption of glucose metabolism but also suggests oxidative stress in LN patients. The elevated levels of UDPGA and xylitol indicate potential blockage in the downstream pentose phosphate pathway, a critical provider of reductants in the body^26^. Furthermore, we identified significant decreases in the serum concentrations of numerous amino acids in LN patients, including glycine, histidine, lysine, phenylalanine, serine, threonine, tryptophan, valine, ornithine, and tyrosine. Concurrently, studies have reported a substantial increase in urea content in LN patients' serum, with urea being a primary metabolite in amino acid energy metabolism. This suggests heightened energy metabolism derived from amino acids, contrasting with weakened fat and sugar-derived energy metabolism in LN patients. Notably, the concentration of valine, a branched-chain amino acid (BCAA), also significantly decreased in LN serum. BCAAs serve as substrates for energy and gluconeogenesis, intimately linked to energy metabolism. Thus, the reduction in valine concentration supports the notion that amino acids play a role in the energy metabolism of LN patients.

A notable reduction in serum glycine is observed in various metabolic-related diseases, particularly in obese patients^27^. Glycine undergoes primary catabolism in the liver, with a considerable amount further metabolized in the intestine. Literature reports emphasize glycine's crucial role as an essential amino acid supporting optimal intestinal flora growth^28^. The marked decrease in serum glycine concentration among LN patients may indicate an imbalance in intestinal microbial metabolism. β-alanine is metabolized by Escherichia coli and Corynebacterium glutamicum in the human intestine. Altered alanine levels may be linked to the metabolic disturbances in LN patients' intestinal microorganisms. Our investigation uncovered a significant decline in tryptophan concentration in the serum of LN patients, potentially associated with the disrupted microbial metabolism in the intestine. Other studies reported elevated concentrations of xanthuric acid and kynurenic acid, metabolites of tryptophan through the kynurenine pathway, in stool samples of SLE patients, suggesting intensified tryptophan metabolism by intestinal flora^29^. The kynurenine pathway is closely tied to excessive immune activation^30^. Simultaneously, we noted a significant decrease in serum tyrosine concentration among LN patients. The concurrent decline in tryptophan and tyrosine levels may imply heightened aromatic amino acid decarboxylase (AADC) activity, converting them into trace amine neurotransmitters^23^. These shifts in serum amino acid levels may influence mood and be associated with depression, as LN patients often exhibit unexplained pathological manifestations in the central nervous system^31^.

In this study, the serum creatinine levels notably rose in LN patients, signifying a potential association with kidney injury. Guanosine acid exhibits superior immune dominance compared to other nucleotides, and the anti-guanosine acid antibody (anti-G) is directly implicated in the pathological processes of SLE^32^. The serum of LN patients manifests a markedly elevated concentration of anti-G in comparison to healthy individuals, wherein the anti-G level is indicative of the extent of renal damage in LN patients^33^. Additionally, our investigation revealed a significant reduction in guanosine acid levels in the serum of LN patients, potentially attributed to the heightened anti-G levels in LN. Similar to anti-G, the guanosine acid level may also serve as a marker for assessing the degree of renal damage in LN patients.

Elevated levels of glutamate and glutamine were observed in the serum of LN patients. Glutamate, a metabolite of glutamine, serves as a crucial synthetic precursor for glutathione (GSH), a vital antioxidant in human cells. Previous reports note insufficient GSH content and reduced activity of cysteine ligase, the enzyme catalyzing GSH production, in SLE patients^34^. The marked increase in serum glutamate and glutamine levels in LN patients suggests a disrupted GSH synthesis pathway, leading to heightened oxidative stress—an affirmation of prior research findings^35^.

Furthermore, this study revealed a significant decrease in the serum content of histidine among LN patients. Histidine, a key antioxidant in the human body, plays a role in neutralizing reactive oxygen species generated during acute inflammatory responses. The diminished serum histidine concentration may be closely linked to oxidative stress, inflammation, and protein energy consumption^36^.

Reports indicate that, in response to oxidative stress damage in SLE patients, the peroxidase and oxidoreductase activities of immunoglobulins—utilizing specific harmful compounds as substrates in serum—are significantly elevated compared to those in healthy individuals^37^. Hydroquinone serves as one of its specific oxidative substrates. This study revealed a notable decrease in serum hydroquinone levels in the LN group, likely attributed to heightened enzyme activity in LN patients. This increase is considered a protective mechanism against harmful compounds in LN patients. Additionally, a significant reduction in serum ornithine content was observed in LN patients, with the alteration closely linked to the arginine-ornithine metabolic pathway. Arginine undergoes two primary metabolic pathways—ornithine metabolism by arginase and the metabolism of nitric oxide (NO) and citrulline through NO synthase (NOS). The hyperactivity of NOS, leading to oxidative stress, is considered one of the pathogenic mechanisms in SLE^38^. The decrease in ornithine levels may reflect NOS hyperactivity in LN patients, significantly diminishing ornithine production under the pathological background of competitive arginine catabolism^39^. Furthermore, a significant increase in palmitic acid levels was noted in the serum of LN patients. Palmitic acid is known to elevate significantly in obese patients and can induce inflammatory reactions in vivo by activating toll-like receptors (TLR)^40^. Additionally, phospholipids, diacylglycerol, and ceramides—intracellular metabolites of palmitic acid—can activate TLR4 through lipopolysaccharide, triggering an inflammatory response. These metabolites contribute to increased reactive oxygen species (ROS) production, ultimately exacerbating the oxidative stress state in vivo^41^.

Simultaneously, a significant decrease in the concentration of piceatannol was noted in the serum of LN patients. Piceatannol possesses the ability to inhibit nuclear factor kappa-B, thereby mitigating inflammation and oxidative stress induced by palmitic acid in the human body^42^. Consequently, the elevation in serum palmitic acid concentration and the reduction in piceatannol concentration among LN patients may be closely associated with inflammatory responses and oxidative stress in these individuals. Ceramide (Cer) is recognized as a central metabolic molecule in the sphingolipid metabolic pathway. Its primary production occurs through the catabolism of sphingomyelin (SM) in the human body, a process regulated by sphingomyelinase (SMase). Reactive oxygen species (ROS) can stimulate SMase activation, leading to an increased metabolic production of Cer. Ceramide, in turn, enhances ROS production, creating a positive feedback loop^43^. This mechanism likely explains the significant increase in serum Cer concentration and the notable decrease in SM levels observed in LN patients.

Cystathionine, an irreversible metabolite of homocysteine, has been implicated in the context of atherosclerotic cardiovascular disease (ASCVD) in SLE patients^44^. This observation may elucidate the significantly higher concentration of homocysteine in LN patients compared to healthy controls. D-Erythro-Sphingosine, synthesized from serine metabolism, has been established to inhibit the function of protein kinase C (PKC)^45^. Previous studies indicate decreased PKC content and significantly reduced functional activity in SLE patients^21^. In the present study, the substantial increase in d-Erythro-Sphingosine levels in the serum of LN patients, known for its inhibitory action on PKC, suggests its potential as a biomarker for the pathological processes of LN. The observed significant elevation in serum hydroxyproline levels in LN patients is noteworthy. Hydroxyproline, a major component of collagen tissue, serves as an indicator of in vivo collagen catabolism, reflecting the breakdown of connective tissue^46^. The increased hydroxyproline level directly mirrors the destruction of connective tissue, aligning with LN's autoimmune nature characterized by connective tissue damage. Consequently, hydroxyproline levels may provide insights into the degree of pathological destruction. Moreover, nearly all glycerol phospholipid metabolites demonstrated a significant decrease in the serum of LN patients, including phosphatidylserine (PS), phosphatidylinositol (PI), phosphatidylglycerol (PG), phosphatidylethanolamine (PE), phosphatidylcholine (PC), and phosphatidic acid (PA). This decrease is likely associated with secondary antiphospholipid syndrome (APS) in LN. APS, a common complication of SLE, manifests with recurrent arteriovenous thrombosis and spontaneous abortion as primary clinical features^47^.

This study has identified numerous biomarkers displaying significant differences between the LN group and the healthy population. It has enriched the LN metabolite database, offering increased prospects for early LN diagnosis. The analysis of a patient's metabolites can unveil early disease abnormalities, facilitating the selection of the most effective treatment. For instance, specific metabolites may manifest abnormalities prior to an official LN diagnosis, allowing for early detection. Periodic monitoring of a patient's metabolites during treatment provides real-time insights into treatment efficacy and allows for prompt adjustments to the treatment plan. Moreover, it aids in predicting the disease's development trend, providing doctors with a more informed basis for decision-making. Concurrently, several potential biomarkers detected in this study align with those identified in prior studies, reinforcing the repeatability of metabolomics research. These consistently validated potential biomarkers merit further investigation. However, we acknowledge the limitations of our research. One such limitation is the geographic bias in our sample population, primarily sourced from southwest China. This bias may restrict the generalizability of our findings to other regions within China or globally. Future research endeavors should contemplate recruiting participants from a more diverse geographical spectrum to ensure sample representativeness and enhance the applicability of our findings. Another limitation pertains to the use of serum as the sample type, which may not comprehensively capture the immune response or other relevant biological processes associated with LN. Subsequent studies could explore alternative sample types, such as urine or kidney biopsy samples, to gain a more comprehensive understanding of disease pathogenesis and progression. To address these limitations, future research should aim to diversify the sample population by including participants from different regions and ethnic backgrounds. Employing multiple sample types can provide a more comprehensive immune response profile. Additionally, validation studies in independent cohorts are imperative to confirm the reliability and generalizability of our findings.

## Conclusions

In conclusion, this study leveraged GC/MS and LC/MS for metabolomic analysis of the serum from Chinese LN patients, aiming to attain a more comprehensive understanding of metabolomic characteristics and identify numerous potential biomarkers with significant differences. Employing multivariate data analysis and metabolic pathway analysis, we identified 41 metabolites that hold promise as early diagnostic markers for LN. These metabolites primarily pertain to immune regulation, energy metabolism, intestinal microbial metabolism, renal damage, and oxidative stress. Notably, some of these potential biomarkers have received consistent validation across various metabolic studies. To establish the clinical utility of these findings, further validation experiments are imperative. Subsequent studies should aim to confirm the widespread applicability of these biomarkers in clinical settings.

## Data Availability

The datasets used and/or analysed during the current study are available from the corresponding author on reasonable request.

## References

[1] Kiriakidou M, Ching CL (2020). Systemic lupus erythematosus. Ann. Intern. Med..

[2] Weinstein A, Alexander RV, Zack DJ (2021). A review of complement activation in SLE. Curr. Rheumatol. Rep..

[3] Yu F, Haas M, Glassock R, Zhao MH (2017). Redefining lupus nephritis: Clinical implications of pathophysiologic subtypes. Nat. Rev. Nephrol..

[4] Owen KA, Grammer AC, Lipsky PE (2022). Deconvoluting the heterogeneity of SLE: The contribution of ancestry. J. Allergy Clin. Immunol..

[5] Morais SA, Isenberg DA (2017). A study of the influence of ethnicity on serology and clinical features in lupus. Lupus.

[6] Piga M, Arnaud L (2021). The main challenges in systemic lupus erythematosus: Where do we stand?. J. Clin. Med..

[7] Davies JC, Midgley A, Carlsson E (2020). Urine and serum S100A8/A9 and S100A12 associate with active lupus nephritis and may predict response to rituximab treatment. RMD Open..

[8] Alseekh S, Aharoni A, Brotman Y, Contrepois K, D'Auria J, Ewald J (2021). Mass spectrometry-based metabolomics: A guide for annotation, quantification and best reporting practices. Nat. Methods.

[9] Nielsen J (2017). Systems biology of metabolism: A driver for developing personalized and precision medicine. Cell Metab..

[10] Trifonova OP, Maslov DL, Balashova EE, Lokhov PG (2021). Mass spectrometry-based metabolomics diagnostics—Myth or reality?. Expert Rev. Proteomics.

[11] Zhang A, Sun H, Wang P, Han Y, Wang X (2012). Modern analytical techniques in metabolomics analysis. Analyst.

[12] Zeki ÖC, Eylem CC, Reçber T, Kır S, Nemutlu E (2020). Integration of GC-MS and LC-MS for untargeted metabolomics profiling. J. Pharm. Biomed. Anal..

[13] Di Minno A, Gelzo M, Stornaiuolo M, Ruoppolo M, Castaldo G (2021). The evolving landscape of untargeted metabolomics. Nutr. Metab. Cardiovasc. Dis..

[14] Hochberg MC (1997). Updating the American College of Rheumatology revised criteria for the classification of systemic lupus erythematosus. Arthritis Rheum..

[15] Wang W, Kou J, Zhang M, Wang T, Li W, Wang Y, Xie Q, Wei M (2023). A metabonomic study to explore potential markers of asymptomatic hyperuricemia and acute gouty arthritis. J. Orthop. Surg. Res..

[16] Vimal A, Kumar A (2018). l-Asparaginase: A feasible therapeutic molecule for multiple diseases. 3 Biotech..

[17] Pacheco R, Contreras F, Zouali M (2014). The dopaminergic system in autoimmune diseases. Front. Immunol..

[18] Ma GJ, Qian X, Chen Z, Chen SS, Zhang AQ (2021). Application of multigroup technology in non-small-cell lung cancer with QI stagnation and blood stasis syndrome. Evid. Based Complement. Alternat. Med..

[19] Hagihara M, Nagatsu T, Ohhashi M, Miura T (1990). Concentrations of neopterin and biopterin in serum from patients with rheumatoid arthritis or systemic lupus erythematosus and in synovial fluid from patients with rheumatoid or osteoarthritis. Clin. Chem..

[20] Schmitz K, Trautmann S, Hahnefeld L, Fischer C, Schreiber Y, Wilken-Schmitz A (2021). Sapropterin (BH4) aggravates autoimmune encephalomyelitis in mice. Neurotherapeutics.

[21] Wu T, Xie C, Han J, Ye Y, Weiel J, Li Q (2012). Metabolic disturbances associated with systemic lupus erythematosus. PLoS ONE.

[22] Szabó MZ, Szodoray P, Kiss E (2017). Dyslipidemia in systemic lupus erythematosus. Immunol. Res..

[23] Bengtsson AA, Trygg J, Wuttge DM, Sturfelt G, Theander E, Donten M (2016). Metabolic profiling of systemic lupus erythematosus and comparison with primary Sjögren’s syndrome and systemic sclerosis. PLoS ONE.

[24] Alonso A, Julià A, Vinaixa M, Domènech E, Fernández-Nebro A, Cañete JD (2016). Urine metabolome profiling of immune-mediated inflammatory diseases. BMC Med..

[25] Karlstad MD, DeMichele SJ, Bistrian BR, Blackburn GL (1991). Effect of total parenteral nutrition with xylitol on protein and energy metabolism in thermally injured rats. JPEN J. Parenter. Enteral Nutr..

[26] Stincone A, Prigione A, Cramer T, Wamelink MM, Campbell K, Cheung E (2015). The return of metabolism: Biochemistry and physiology of the pentose phosphate pathway. Biol. Rev. Camb. Philos. Soc..

[27] Guasch-Ferré M, Hruby A, Toledo E, Clish CB, Martínez-González MA, Salas-Salvadó J (2016). Metabolomics in prediabetes and diabetes: A systematic review and meta-analysis. Diabetes Care.

[28] Dai ZL, Wu G, Zhu WY (2011). Amino acid metabolism in intestinal bacteria: Links between gut ecology and host health. Front. Biosci. (Landmark Ed.).

[29] Zhang Q, Yin X, Wang H, Wu X, Li X, Li Y (2019). Fecal metabolomics and potential biomarkers for systemic lupus erythematosus. Front. Immunol..

[30] Chen Y, Guillemin GJ (2009). Kynurenine pathway metabolites in humans: Disease and healthy states. Int. J. Tryptophan Res..

[31] Jeltsch-David H, Muller S (2014). Neuropsychiatric systemic lupus erythematosus: Pathogenesis and biomarkers. Nat. Rev. Neurol..

[32] Stollar BD, Borel Y (1976). Nucleoside specificity in the carrier IgG-dependent induction of tolerance. J. Immunol..

[33] Colburn KK, Green LM, Wong AK (2001). Circulating antibodies to guanosine in systemic lupus erythematosus: Correlation with nephritis and polyserositis by acute and longitudinal analyses. Lupus.

[34] Gergely P, Grossman C, Niland B, Puskas F, Neupane H, Allam F (2002). Mitochondrial hyperpolarization and ATP depletion in patients with systemic lupus erythematosus. Arthritis Rheum..

[35] Song W, Yuan J, Zhang Z, Li L, Hu L (2014). Altered glutamate cysteine ligase activity in peripheral blood mononuclear cells from patients with systemic lupus erythematosus. Exp. Ther. Med..

[36] Kumar A, Bala L, Kalita J, Misra UK, Singh RL, Khetrapal CL (2010). Metabolomic analysis of serum by (1) H NMR spectroscopy in amyotrophic lateral sclerosis. Clin. Chim. Acta.

[37] Tolmacheva AS, Buneva VN, Nevinsky GA (2019). Substrate specificity of IgGs with peroxidase and oxidoreductase activities from sera of patients with systemic lupus erythematosus and multiple sclerosis. J. Mol. Recogn..

[38] Pan L, Yang S, Wang J, Xu M, Wang S, Yi H (2020). Inducible nitric oxide synthase and systemic lupus erythematosus: A systematic review and meta-analysis. BMC Immunol..

[39] Feng R, Luo C, Li C, Du S, Okekunle AP, Li Y (2017). Free fatty acids profile among lean, overweight and obese non-alcoholic fatty liver disease patients: A case-control study. Lipids Health Dis..

[40] Snodgrass RG, Huang S, Choi IW, Rutledge JC, Hwang DH (2013). Inflammasome-mediated secretion of IL-1β in human monocytes through TLR2 activation; modulation by dietary fatty acids. J. Immunol..

[41] Maloney E, Sweet IR, Hockenbery DM, Pham M, Rizzo NO, Tateya S (2009). Activation of NF-kappaB by palmitate in endothelial cells: A key role for NADPH oxidase-derived superoxide in response to TLR4 activation. Arterioscler. Thromb. Vasc. Biol..

[42] Jeong SO, Son Y, Lee JH, Cheong YK, Park SH, Chung HT (2015). Resveratrol analog piceatannol restores the palmitic acid-induced impairment of insulin signaling and production of endothelial nitric oxide via activation of anti-inflammatory and antioxidative heme oxygenase-1 in human endothelial cells. Mol. Med. Rep..

[43] Andrieu-Abadie N, Gouazé V, Salvayre R, Levade T (2001). Ceramide in apoptosis signaling: Relationship with oxidative stress. Free Radic. Biol. Med..

[44] Summers CM, Cucchiara AJ, Nackos E, Hammons AL, Mohr E, Whitehead AS (2008). Functional polymorphisms of folate-metabolizing enzymes in relation to homocysteine concentrations in systemic lupus erythematosus. J. Rheumatol..

[45] Yan B, Huang J, Zhang C, Hu X, Gao M, Shi A (2016). Serum metabolomic profiling in patients with systemic lupus erythematosus by GC/MS. Mod. Rheumatol..

[46] Li P, Wu G (2018). Roles of dietary glycine, proline, and hydroxyproline in collagen synthesis and animal growth. Amino Acids.

[47] Ünlü O, Zuily S, Erkan D (2016). The clinical significance of antiphospholipid antibodies in systemic lupus erythematosus. Eur. J. Rheumatol..

